# Amniotic Membrane-Derived Mesenchymal Cells and Their Conditioned Media: Potential Candidates for Uterine Regenerative Therapy in the Horse

**DOI:** 10.1371/journal.pone.0111324

**Published:** 2014-10-31

**Authors:** Bruna Corradetti, Alessio Correani, Alessio Romaldini, Maria Giovanna Marini, Davide Bizzaro, Claudia Perrini, Fausto Cremonesi, Anna Lange-Consiglio

**Affiliations:** 1 Department of Life and Environmental Sciences, Università Politecnica delle Marche, Ancona, Italy; 2 Large Animal Hospital, Reproduction Unit, Università degli Studi di Milano, Lodi, Italy; 3 Department of Veterinary Science for Animal Health, Production and Food Safety, Università degli Studi di Milano, Milan, Italy; University of Quebec at Trois-Rivieres, Canada

## Abstract

Amniotic membrane-derived mesenchymal cells (AMCs) are considered suitable candidates for a variety of cell-based applications. In view of cell therapy application in uterine pathologies, we studied AMCs in comparison to cells isolated from the endometrium of mares at diestrus (EDCs) being the endometrium during diestrus and early pregnancy similar from a hormonal standpoint. In particular, we demonstrated that amnion tissue fragments (AM) shares the same transcriptional profile with endometrial tissue fragments (ED), expressing genes involved in early pregnancy (*AbdB-like Hoxa* genes), pre-implantantion conceptus development (*Erα, PR*, *PGRMC1* and *mPR*) and their regulators (*Wnt7a*, *Wnt4a*). Soon after the isolation, only AMCs express *Wnt4a* and *Wnt7a*. Interestingly, the expression levels of prostaglandin-endoperoxide synthase 2 (*PTGS2*) were found greater in AM and AMCs than their endometrial counterparts thus confirming the role of AMCs as mediators of inflammation. The expression of nuclear progesterone receptor (PR), membrane-bound intracellular progesterone receptor component 1 (*PGRMC1*) and membrane-bound intracellular progesterone receptor (*mPR*), known to lead to improved endometrial receptivity, was maintained in AMCs over 5 passages *in vitro* when the media was supplemented with progesterone. To further explore the potential of AMCs in endometrial regeneration, their capacity to support resident cell proliferation was assessed by co-culturing them with EDCs in a transwell system or culturing in the presence of AMC-conditioned medium (AMC-CM). A significant increase in EDC proliferation rate exhibited the crucial role of soluble factors as mediators of stem cells action. The present investigation revealed that AMCs, as well as their derived conditioned media, have the potential to improve endometrial cell replenishment when low proliferation is associated to pregnancy failure. These findings make AMCs suitable candidates for the treatment of endometrosis in mares.

## Introduction

Embryo-maternal communication is a pre-requisite for successful implantation that facilitates the establishment, recognition, and maintenance of pregnancy. Studies of early pregnancy in different mammalian species have shown that the majority of embryo losses occurs during the pre-implantation phase: in the horse, this phase is very critical [1], [2]. During this period, the conceptus (the embryo and the associated extra-embryonic membranes) interacts with the uterine environment via paracrine signaling to coordinate attachment and implantation. Disruption of the integrity of the endometrial tissue and of the capacity to support its functions are the responsible factors in determining a subfertile phenotype [3]. As such, the development of chronic endometritis followed by degenerative and abnormal changes of the endometrium, like fibrosis (scarring) around the glands, might inhibit the regeneration of traumatized endometrium. The damage then leads to an impairment of the progenitor cell ratio that reduces the endometrial tissue's ability to regenerate and thus future foaling rates as well. This mainly results in the inability of the tissue to support embryo implantation.

Given the potential role of these cells have in remodeling the endometrium, it may be reasonable to suggest cell therapies based on mesenchymal stem cells (MSCs) as suitable strategies to restore and maintain normal function in damaged reproductive tissues.

With this in mind, we recently retrieved MSCs from extra-fetal tissues in the equine species [4]. For the first time, we compared the proliferative and differentiative potential of amniotic membrane-derived MSCs (AMCs) with bone marrow derived MSCs (BM-MSCs). together with their possible application in the treatment of horse tendon injuries [5], [6]. We next demonstrated the potential immunomodulatory properties of AMCs and their conditioned medium (AMC-CM) *in vitro*, and proved the efficacy of AMC-CM in the treatment of spontaneous horse tendon and ligament injuries *in vivo* [7]. Data obtained in this study demonstrated the crucial role of soluble factors in inhibiting peripheral blood mononucleated cell proliferation *in vitro* and in improving healing *in vivo*. Taken together, the AMCs features described so far by our group suggest this cell type as the most suitable for regenerative medicine approaches [5] and its derived conditioned media (CM) as a novel, cell-free therapeutic product in regenerative medicine.

For these reasons, the aim of the present study was to better understand the nature of AMCs, so that we may exploit their potential role in uterine replacement therapy. AMCs were further characterized for the expression of early pregnancy-associated genes in view of their potential application in endometrial regeneration or in stimulating the proper preparation of the endometrium for embryo implantation.

The AbdB-like Hoxa gene (Hoxa9) and the wingless type genes (Wnt7a, Wnt4a), which influence pre-implantantion conceptus development, the classical oestrogen (ERα and ERβ) and progesterone (PR) receptors, and the more recently characterized membrane-bound intracellular progesterone receptors (PGRMC1 and mPR), were evaluated on amniotic membrane fragment (AM) or AMCs (at different passages) in comparison to endometrial tissue fragments (ED) or isolated endometrial cells (EDCs) at diestrus. We also looked at the expression of other genes, including the cyclooxygenase prostaglandin E2 synthase (*PTGS2*), forkhead box 01 (*FOXO1*), serum/glucocorticoid regulated kinase 1 (*SGK1*), and tumor protein p53 inducible protein3 (*TP53*) whose transcriptional activity has been associated with luteo-protective action [8], protection of the feto-maternal unit against oxidative damage [9], improved reproductive outcome [10], and epithelial ion transport and cell survival responses [11] during early pregnancy, respectively. Since the endometrial stromal and epithelial cells proliferation and differentiation through the period of embryo preimplantation are controlled by ovarian steroid hormones (progesterone and estrogen) [12], [13], we aimed to recreate the endometrial environment, thus culturing AMCs for different passages in the presence of progesterone.

We further hypothesized that AMCs might induce tissue-resident endometrial cells to proliferate or, alternatively, to modulate the local immune response and promote endometrial regeneration. MSCs, in general, are believed to play their role as mediators of tissue repair through the release of paracrine factors [14]. To test this hypothesis EDCs were cultured either with AMCs in a transwell system or with their conditioned media, in order to evaluate the capability of AMCs to increase the proliferative potential of endometrial cells.

## Materials and Methods

### Materials

Samples were collected from horses slaughtered in a national slaughterhouse (Titbit Srl, Bagnolo in Piano, Reggio Emilia, Italy) with legal regulations.

Chemicals were obtained from Sigma-Aldrich Chemical (Milan, Italy) unless otherwise specified, and tissue culture plastic dishes were purchased from Euroclone (Milan, Italy).

### Tissue collection and isolation

Three amniotic membranes were obtained at term of normal pregnancies after vaginal delivery from three mares. Clean collected portions of allanto-amnion were kept at 4°C in PBS with 100 U/ml penicillin, 100 µg/ml streptomycin and 4 µg/ml amphotericin B and were processed within 8 h. The amniotic membrane was stripped from the overlying allantois and cut into small pieces (about 9 cm^2^ each) before starting the enzymatic digestion. Endometrial tissues were collected from 3 different horses aged between 8 and 10 years, after euthanasia unrelated to our experiments. Endometrial samples were obtained during the reproductive season from normal-cycling mares at diestrus (early-mid luteal phase). Before euthanasia, 5 ml of blood were collected in heparinized tubes from all mares. After centrifugation, plasma was separated, kept refrigerated and immediately transported to the laboratory for progesterone determination by a quantitative Enzyme Linked Fluorescent Assay (ELFA) based on the MiniVidas (Biomerieux, Firenze, Italy) technology. According to the manufacturer, the measurement range of the assay varied from 0.25 to 80 ng/ml with an intra-assay variability of 4.12% and an inter-assay variability of 6.32%.

Only uteri belonging to mares with an obvious corpus luteum on the ovary and progesterone levels between 6 to 20 ng/ml, indicative of the early/mid diestral phase of the estrous cycle [15], were used for endometrial fragment collection and ensuing cell culture.

Tissue fragments for RNA isolation were immediately immersed in RNA Later solution, whereas those destined for cell isolation and expansion procedure were kept at 4°C in saline solution supplemented with 4 µg/ml amphotericin B, 100 IU/ml penicillin and 100 µg/ml streptomycin and were processed within 8 h.

### Cell isolation

Amniotic membrane-derived mesenchymal cells were isolated as previously reported [4]. Briefly, amnion fragments were incubated for 9 min at 38.5°C in PBS containing 2.4 U/mL dispase (Becton Dickinson, Milan, Italy). After a resting period (5–10 min) at room temperature in high-glucose Dulbecco's modified Eagle's medium (HG-DMEM; EuroClone, Milan, Italy), supplemented with 10% heat inactivated fetal bovine serum (FBS) and 2 mM L-glutamine, the fragments were digested with 0.93 mg/mL collagenase type I and 20 mg/mL DNAse (Roche, Mannheim, Germany) for approximately 3 h at 37°C. The amnion fragments were then removed, and mobilized cells were passed through a 100 µm cell strainer before being collected by centrifugation at 200×g for 10 min.

Endometrial cells from diestrus mare's uteri were obtained according to the protocol described by Donofrio et al. [16] and slightly modified for equine cells. Briefly, the endometrium was digested in sterile filtered Hank's buffered salt solution supplemented with 2 mg/ml collagenase II, 4 mg/ml bovine serum albumine, and 0.4 mg/ml DNase I for 90 minutes at 38.5°C in a shaking bath. Cells were then filtered through a membrane with a pore size of 80 µm, centrifuged at 200×g for 10 minutes, and then washed twice in PBS. This protocol allowed for the isolation of the endometrial stromal portion.

Fibroblasts (used as a control for AMCs) were isolated from skin specimens collected from three horses after euthanasia unrelated to our experiments. Samples (2 mm^3^) were taken by excision and fragments of approximately 1 mm were incubated with 40 mg/ml collagenase IV for 3 h at 38.5°C. After digestion, cells were washed and allowed to grow to confluence in 60-mm culture dishes.

Before seeding, the primary cultures (P0) obtained from the amnion, endometrium and skin were counted using a Burker chamber with the trypan blue dye exclusion assay.

### Cell expansion

Endometrial and fibroblast cells cultures were established in HG-DMEM supplemented with 10% FBS, penicillin (100 UI/ml)-streptomycin (100 µg/ml), 0.25 µg/ml amphotericin B and 2 mM L-glutamine. Medium was supplemented with 10 ng/ml epidermal growth factor (EGF) for AMC cultures.

To remove non-adherent cells, the medium was replaced for the first time after 72 h, and then changed either twice per week thereafter or according to the experiment requirements. For maintenance of cultures, cells were plated in flasks of 25 cm^2^ at the density of 1×10^5^ cells/cm^2^ and incubated at 38.5°C in a humidified atmosphere with 5% CO_2_. Adherent cells were detached with 0.05% trypsin-EDTA just prior to reaching confluence (80%) and then reseeded for culture maintenance at the density of 1×10^4^ cells/cm^2^.

Molecular characterization of EDCs was performed only at P0 as *de facto* control for gene expression, whereas AMCs were studied from P0 to P5.

### Preparation of conditioned medium

AMCs and fibroblasts, at P3, were plated in 24-well plates at a density of 1×10^5^ cells/well in DMEM standard complete medium. To obtain AMC-CM and fibroblast-CM, cells were cultured for 5 days at 38.5°C in a humidified atmosphere with 5% CO_2_. Supernatants from each plate were then collected, pooled, centrifuged at 700×g, filtered (0.2 µm), and stored at −80°C. This procedure was performed for cells obtained from three different placentas and from three different samples of skin.

The collected supernatants were lyophilized and stored at 4°C until use, at which point they were dissolved in sterile cell culture water to one-quarter of the initial volume.

### Molecular characterization

Total RNA was extracted from tissues (AM and ED) and cells (AMCs and EDCs) immediately after isolation (P0) using TRI Reagent® Solution (Life Technologies, Monza, Italy). Total RNA was also extracted from AMCs at different passages (P1, P3 and P5) and after culture in the presence of progesterone. Samples were then treated with DNase in order to avoid DNA contamination. RNA concentration and purity were measured by Nanodrop Spectrophotometer (Nanodrop® ND1000). The cDNA was synthesized from total RNA (500 ng) using a Taqman Reverse Transcription reagents kit (Applied Biosystems, Branchburg, NJ). The gene expression evaluation was performed using specific sequences; equine-specific oligonucleotide primers were designed using open source PerlPrimer software v. 1.1.17, based on available NCBI *Equus caballus* sequences or on mammal multi-aligned sequences. Primers were designed across an exon–exon junction in order to avoid genomic DNA amplification and their sequence conditions and the references used are shown in Table 1. Equine glyceraldehyde-3-phosphate dehydrogenase (*GAPDH*) was employed as a reference gene in each sample in order to standardize the results by eliminating variation in mRNA and cDNA quantity and quality.

**Table 1:** Oligonucleotide sequences used for RT-PCR analysis.

REF SEQ	Markers	Forward (5′→3′)	Reverse (5′→3′)	T_Annealing_	bp
XM_001498494.3	Progesteron Receptor (PR)	GTCAGTGGACAGATGCTGTA	CGCCTTGATGAGCTCTCTAA	55°C	255
NM_001081772.1	Estrogen receptor a (ERa)	TCCATGATCAGGTCCACCTTCT	GGTGTCTGTCATCTTGTCCA	55°C	341
XM_001915519.2	Estrogen receptor b (ERb)	TCAGCCTGTTCGACCAAGTG	CCTTGAAGTCGTTGCCAGGA	60°C	194
NM_001256979.1	Membrane-associated progesterone receptor (MPR)	GCCAAGTATCGTTACCGGAG	AAGAGGATCTGGAGCGTGTG	55°C	173
XM_001914705.2	Membrane-associated progesterone receptor (PGRMC1)	TCAACGGCAAGGTGTTCGAC	GGCTCTTCCTCATCTGAGTA	58°C	280
XM_003364827.1	Homeobox protein Hox-A9-like (Hoxa9)	ACGCTGGAACTGGAGAAAGA	CTTTCGCTCGGTCCTTATTG	55°C	160
NM_001163856.1	Glyceraldehyde-3-phosphate dehydrogenase (GAPDH)	AGATCAAGAAGGTGGTGAAG	TTGTCATACCAGGAAATGAGC	59°C	178
XM_001501510.2	Wingless-type MMTV integration site family, member 4 (Wnt7a)	TTTCGGATTCCCGCAGTCTC	CATCTGCACCTGCCTCTGAA	55°C	177
XM_001489623.2	Wingless-type MMTV integration site family, member 7A(Wnt7a)	CATGGTCTACCTCCGGATCG	TATGACGATGATGGCGTCGG	55°C	134
XM_005600181.1	Tumor protein p53 inducible protein3 (TP53)	TCGCATTTCAAGCCAAACCG	CTTCCTTCTCCTTCCCGTCG	60°C	110
XM_005601179.1	Forkhead box 01 (FOX01)	ATTGAGCGCTTGGACTGTG	CGTTGTCTTGACACTGTGC	60°C	126
XM_005609749.1	Prostaglandin-endoperoxide synthase 2 (PTGS2)	GATCCTAAGCGAGGTCCAGC	AGGCGCAGTTTATGCTGTCT	60°C	101
XM_001504377	Serum/glucocorticoid regulated kinase 1 (SGK1)	CTGCACTCCCTGAACATCGT	ATGTTCTCCTTGCAGAGCCC	60°C	107

Conventional RT-PCR was performed in a 25 µl final volume with RBC Taq DNA Polymerase (RBC Bioscience) under the following conditions: initial denaturation at 95°C for 2 minutes, 32 cycles at 95°C for 30 seconds (denaturation), 55–60°C for 30 seconds (annealing), 72°C for 1 minute (elongation) and final elongation at 72°C for 7 minutes. For conventional PCR, primers were used at 300 nM final concentrations.

Quantitative PCRs were performed with SYBR green method in a MyiQ iCycler thermal cycler (Biorad). Triplicate PCR reactions were carried out for each sample analyzed. The reactions were set on a strip in a final volume of 25 µl by mixing, for each sample, 1 µl of cDNA, 12.5 µl of 2X concentrated Power Sybr®Green PCR Master Mix (Applied Biosystems) containing SYBR Green as a fluorescent intercalating agent, 0.2 µm forward primer, 0.2 µm of reverse primer and MQ water. PCR efficiencies were tested and found to be close to 1. The thermal profile for all reactions was 10 minutes at 95°C and then 40 cycles of 15 seconds at 95°C, 1 minute at 60°C. Fluorescence monitoring occurred at the end of each cycle. Additional dissociation curve analysis was per- formed and in all cases showed one single peak. The data thus obtained were analyzed using the iQ5 optical system software version 2.0 (BioRad). Gene expression levels found in AM and AMCs were normalized and represented with respect to ED and EDCs, respectively.

### Proliferation studies

#### Growth curves

To obtain growth curves at P1 and P5, AMCs and EDCs were plated at the density of 9×10^3^ cells into six-well tissue culture plates. Every three days, over the 15 days of culture, cells from one well of each plate were detached and counted.

#### Doubling time (DT)

Cell proliferation was determined as previously reported [4]. DT for passages 1–5 was assessed plating 9×10^3^ cells into six-well tissue culture plates. Every 4 days cells were trypsinized, counted and reseeded at the same density. Mean DT was calculated from day 0 to day 4. The DT value was obtained for each passage according to the formula DT  =  CT/CD, where CT represents the culture time and CD  =  log(N_c_/N_o_)/log2 represents the number of cell generations (N_c_ represents the number of cells at confluence, N_o_ represents the number of seeded cells). Data representative of three independent experiments are reported.

#### EDC proliferation in presence of AMCs or AMC-CM

To assess the potential of AMCs to increase EDCs proliferation (evaluated as growth curves and DT) 24-well plates with 0.4 µm-pore transwell inserts were used to physically separate the two cell populations. EDCs were plated at 1×10^4^ cells/well in standard complete medium and AMCs were added at the same density on top of the inserts.

To evaluate the effect of AMC-CM on EDCs proliferation, growth and DT were studied plating 9×10^3^ cells into six-well tissue culture plates. For this experiment, cultures were established in standard complete medium supplemented with 10% AMC-CM. As a control group, EDCs were studied in the presence of either fibroblasts separated with a transwell system or fibroblast-derived CM.

### AMCs culture in the presence of progesterone

To determine whether AMCs would be effective in replacement therapy we cultured them in the presence of progesterone. Cultures at P1 were established in HG-DMEM supplemented with 10% FBS, 10 ng/ml EGF, 1% penicillin (100 UI/ml)/streptomycin (100 µg/ml), 0.25 mg/ml amphotericin B, 2 mM L-glutamine (standard complete medium) and 20 ng/ml of progesterone. For maintenance of the cultures, cells were plated at 1×10^4^ cells/cm^2^ and incubated at 38.5°C in a humidified atmosphere with 5% CO_2_. Just prior to reaching confluence (80%), adherent cells were detached with 0.05% trypsin-EDTA and then reseeded for culture maintenance at the density of 1×10^4^ cells/cm^2^. The medium was changed twice per week. Cells at P5 were the last time point included for the molecular characterization studies.

### Statistical analysis

For proliferation data, statistical analysis was performed using GraphPad Instat Software version 3.00 for Windows (La Jolla, CA, US). Three replicates for each experiment were performed. Results are reported as mean ± standard deviation (SD). One-way analysis of variance (ANOVA) for multiple comparisons by Student-Newman-Keuls multiple comparison tests was used. Differences were considered statistically significant for P values <0.05. For quantitative PCR data, non-parametric tests were used. The Kruskal-Wallis test was used for comparison between multiple groups whereas Mann-Whitney U-test was employed to compare two groups. P<0.05 was considered significant.

## Results

### Cells isolation and expansion

**Figure 1:**
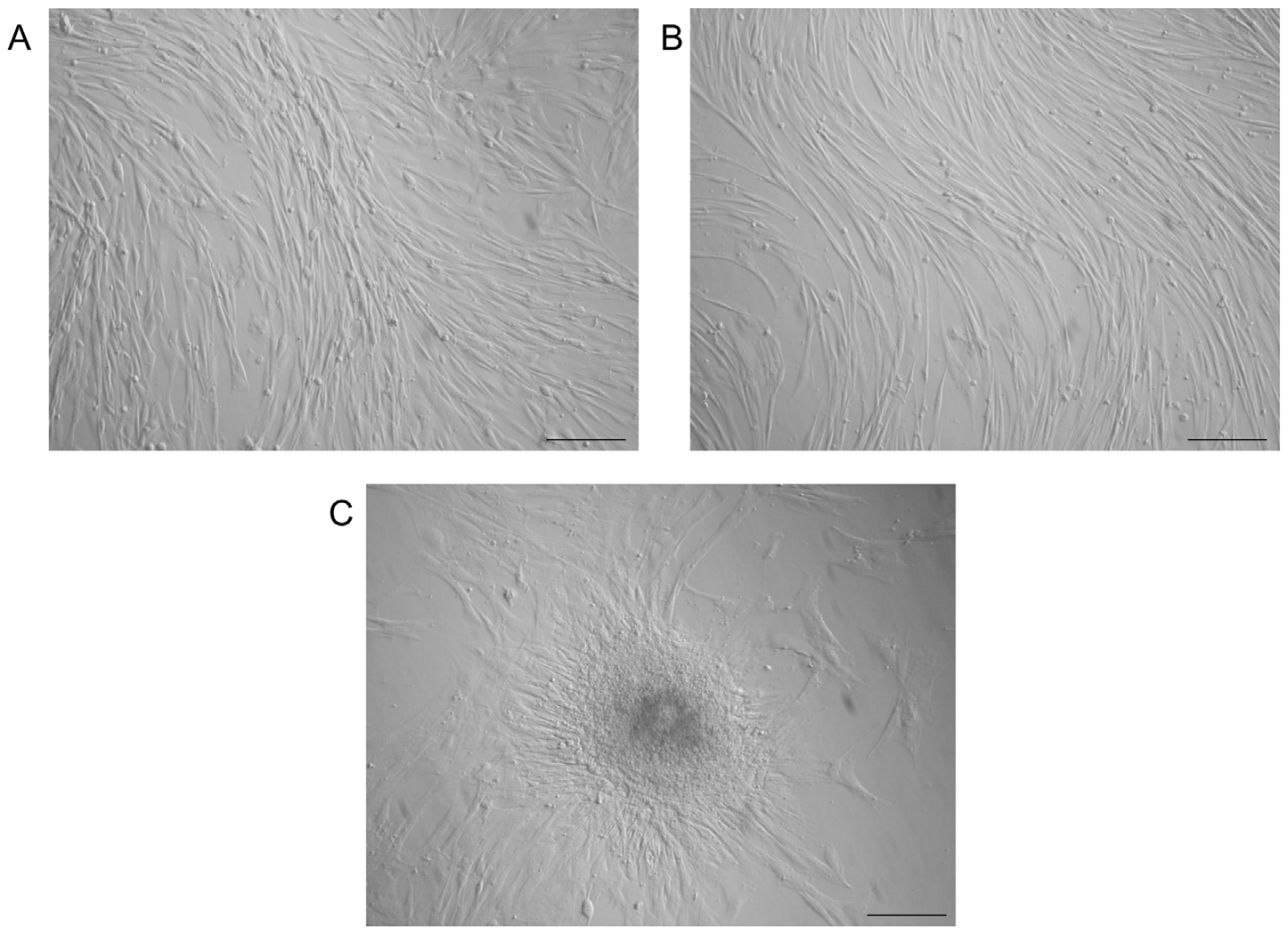
Cell morphology. Monolayer of AMCs (A) and EDCs (B); tridimensional cluster of AMCs (C). Magnification, x 20; scale bar  = 20 µm.

Cells were selected purely on their ability to adhere to plastic. For AMCs, initial viability was >90%, whereas for EDCs it was >80%. All cell lines displayed the typical fibroblast-like morphology (Figs 1A and 1B). AMCs observed at the early stages of culture organised as tridimensional clusters (Fig 1C).

### Molecular characterization

As previously reported, molecular studies demonstrated that at P3 AMCs display a typical mesenchymal stromal phenotype, expressing pluripotent and MSC-specific markers [4]. For further information, see Supplementary Information. When the expression of genes associated to pre-implantantion conceptus development was investigated, conventional RT-PCR analysis demonstrated that *PR, mPR, PGRMC1, Hoxa9, Wnt7a, Wnt4a*, as well as *PTGS2, FOXO1*, *SGK1* and *TP53* were expressed in ED and AM. While *ERα* was expressed by AM and ED, *ERß* was not detected in both tissues.

**Figure 2:**
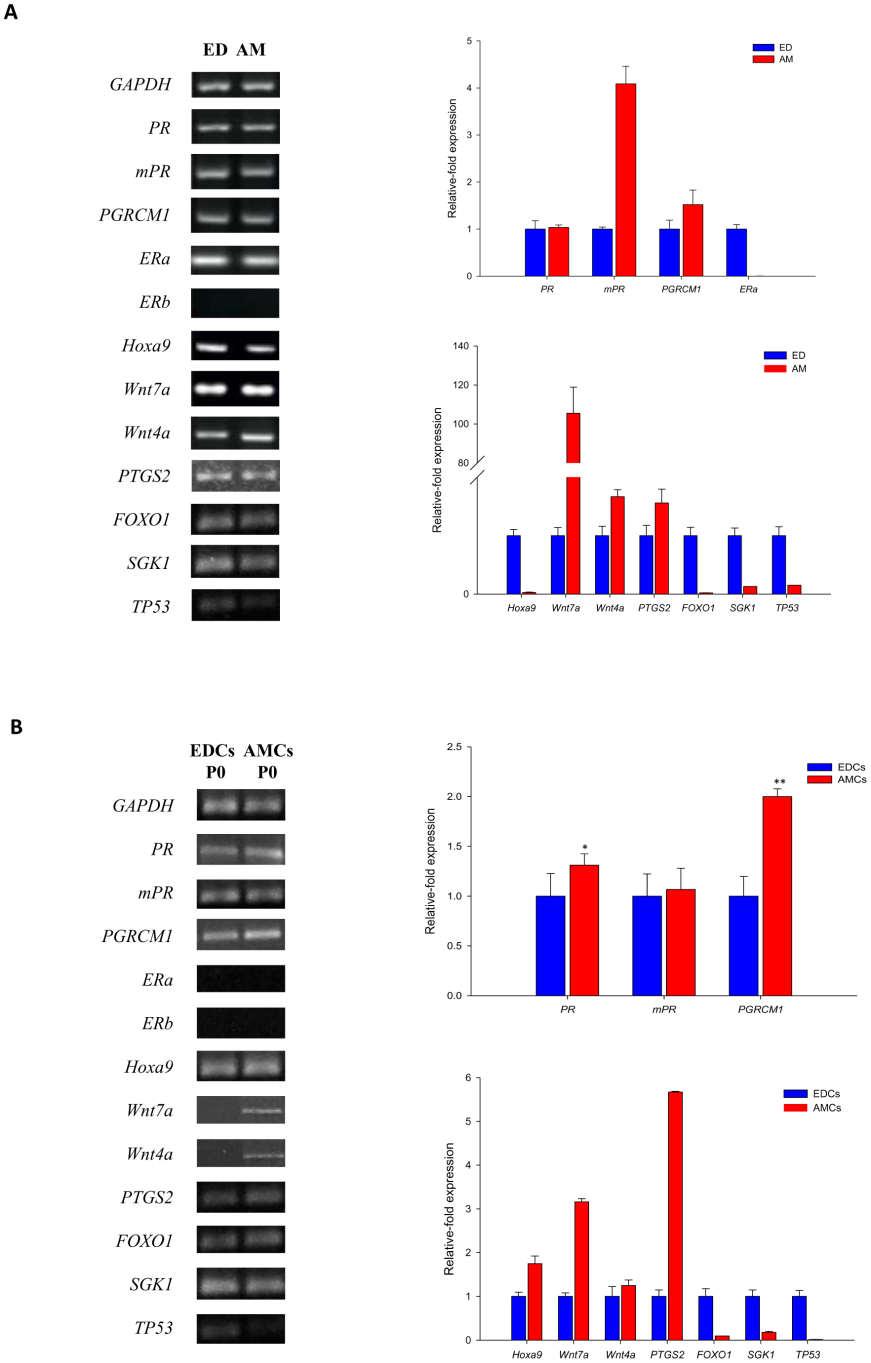
Molecular characterization of endometrial tissue (ED) and amnion (AM) (A), and endometrial cells at diestrus stage (EDCs) and amniotic-derived stem cells (AMCs) (B). Qualitative and quantitative RT–PCR analysis for the expression of genes associated to differentiation of uterine stromal cells during early pregnancy (*Hoxa9*), those influencing pre-implantantion conceptus development (*ERα*, *ERβ*, *PR, PGRMC1* and *mPR*) and their regulators (*Wnt7a*, *Wnt4a*), and prostaglandin E_2_ synthase (*PTGS2*), *FOXO1*, *SGK1*, and *TP53*. *GAPDH* was used as reference gene.

Quantitative RT-PCR showed a statistically significant increase in the expression of *mPR* (4.08±0.37; P<0.01), *PGRCM1* (1.52±0.3; P<0.05), *Wnt7a* (105.41±13.27), *Wnt4a* (1.66±0.11; P<0.05), and *PTGS2* (1.55±0.23; P<0.05) in the comparison of AM to ED (Fig 2A). Although *ERα* expression was demonstrated by conventional RT-PCR, when quantitatively analyzed it was found to be statistically lower when comparing AM to ED.

At P0, right after cell isolation, AMCs expressed all the genes studied with only the exception of *ERα* and *ERß*. When cells from the amniotic membrane and endometrial tissue were quantitatively compared, data showed a significant upregulation in the expression of *PR* (1.3±0.11; P<0.05), *PGRCM1* (2±0.07; P<0.01), *Hoxa9* (1.74±0.17; P<0.05), *Wnt7a* (3.16±0.7; P<0.01), and *PTGS2* (5.66±0.015) in AMCs (Fig 2B).

**Figure 3:**
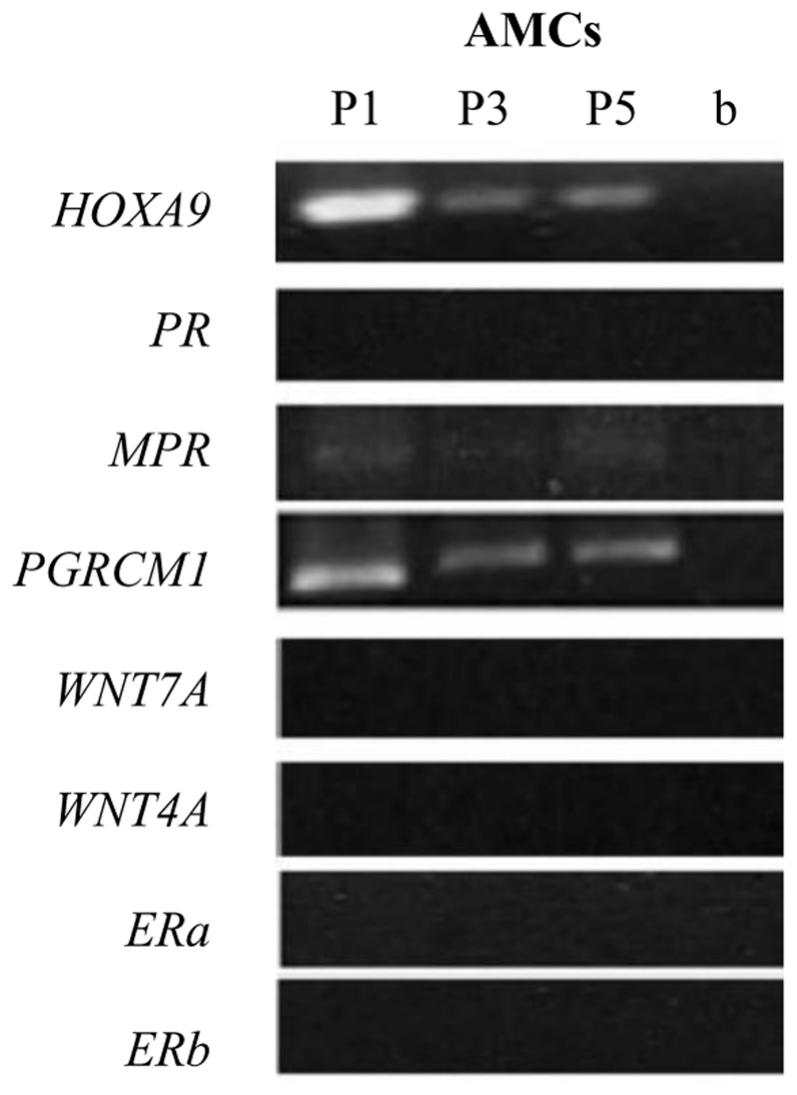
Molecular characterization of AMCs. Conventional RT-PCR analysis of evaluated genes in AMCs after culture with progesterone from passage 1 (P1) to passage (P5).

Despite being qualitatively detected in ED and AM and in cells derived from both sources, the levels of *FOXO1*, *SGK1* and *TP53* found in AM and AMCs were significantly lower in comparison to those observed in ED and EDCs (Fig 2B). No expression for the genes of interest was detected in further passages with only the exception of *Hoxa9* whose mRNA was found at P1 (data not shown). AMCs cultured in the presence of supplemented progesterone, expressed *Hoxa-9*, *PGRMC1* and *mPR* until the last passage studied (Fig 3).

### Proliferation studies

#### Growth curve and DT

**Figure 4:**
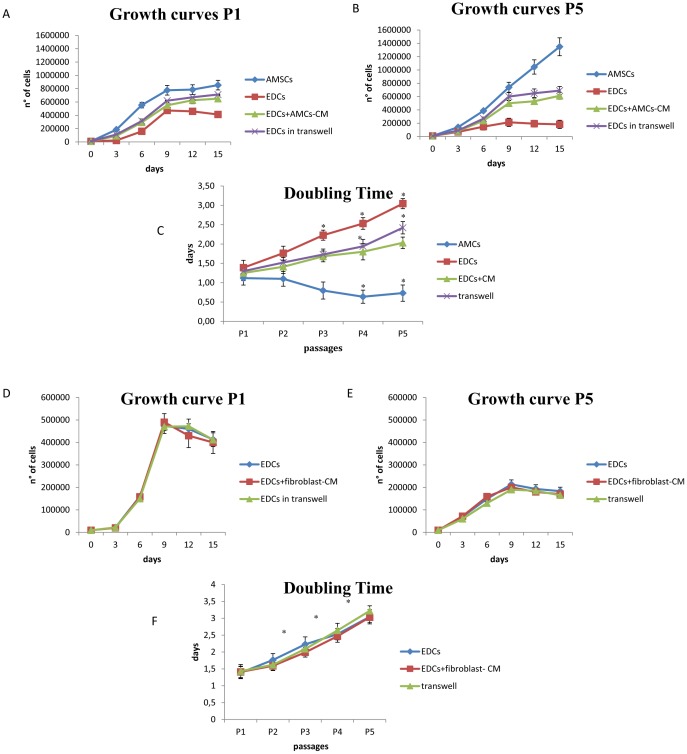
Proliferation studies. Growth curve at P1 (A) and P5 (B) for AMCs and EDCs alone or in co-culture with AMCs by transwell system or by AMC-conditioned medium (AMC-CM). Doubling times at different passages during cell culture for both AMCs and EDCs (C) in different culture conditions. Asterisks represent statistically different doubling times with respect to P1 (*P*<0.01). Growth curve at P1 (D) and P5 (E) for EDCs alone or in co-culture with fibroblast by transwell system or by fibroblast-conditioned medium. Doubling times at different passages during cell culture for EDCs (E) in different culture conditions with fibroblast. Asterisks represent statistically different doubling times with respect to P1 (*P*<0.01).

At P1, EDCs and AMCs demonstrated a growth curve with an initial lag phase of 3 days that decreased at P5 with AMCs growing more rapidly than EDCs during this phase (Figs 4A and 4B). The proliferative potential of EDCs was higher at P1 than at P5 in contrast with the results of AMCs.

The DT values at P1 were similar between the two cell lines (Fig 4C). In EDCs, it significantly increased (P<0.05) toward P5. On the contrary, the greater proliferative ability associated to AMCs was found between P3 and P5, and was further confirmed by the growth curve at passage 5. At each passage, the differences observed between the DT values observed for AMCs and EDCs was statistically significant (P<0.05). The mean DT value for the EDCs was 2.19±0.65 days, while it was 0.88±0.22 days for AMCs.

#### EDCs proliferation in presence of AMCs or AMC-CM

*In vitro* studies demonstrated that co-culture with AMCs significantly increased EDCs proliferation in all passages studied (Figs 4 A, B, C). The mean DT value for EDCs in the transwell experiment (1.78 days) had a decrease of ∼1.23-fold in comparison to baseline EDCs proliferation in standard complete medium (2.19 days; P<0.05 for all comparisons). The culture in the presence of AMC-CM was able to stimulate EDCs proliferation to the same level of that obtained when cells were grown in the transwell system (DT value of 1.63 days with a decrease of ∼1.34-fold). Co-culture with fibroblasts, physically separated through a transwell system or in presence of their CM, did not induce any change in the proliferative potential of EDCs (Figs 4 D, E, F).

## Discussion

As in humans, equine fetal adnexa have been recently suggested as appealing candidates for the derivation of MSCs to be used in cell-based therapies [4], [17]–[26] due to their higher proliferation capacity, longer telomeres, broader differentiation, and extensive proliferative potential in comparison to cells obtained from adult tissues [5], [17], [27].

We previously isolated AMCs from the equine amniotic membranes confirming that these cells share specific characteristics with embryonic and adult stem cells; they express representative mesenchymal (*CD105*, *CD44*, *CD29*, *CD166*) and pluripotent (Tra-1-60, SSEA-4, Oct-4) markers, are highly proliferative and retain high plasticity [4], [5]. We also provided evidence of their superior efficacy in tendon regeneration compared to their bone marrow counterparts when allogeneically transplanted *in vivo* [4], [6].

Taken together, these characteristics make AMCs suitable candidates for endometrial regeneration. To test this hypothesis we further characterized AMCs comparing them to uterine stromal cells collected at diestrus, due to the similarities found in the hormonal profile between early pregnancy and the diestrous stage. Both stages are characterized by high blood progesterone level [28].

Our data demonstrates that at P0, AM and AMCs are similar to ED and EDCs, respectively, for the expression of some endometrial-specific genes. Although our study only assayed a few genes, we selected the ones that are crucial in the process of conceptus-endometrium interaction. In particular, we found the expression of *Wnt4a* statistically higher in AM than ED and with similar results between AMCs and EDCs. This data can be explained with the role that the wingless type genes play in reproductive events: including the establishment and maintenance of pregnancy, implantation, proliferation, and secretion activity of endometrium during the estrous cycle and pregnancy [29]–[32].

According to this, we can hypothesize that in horses *Wnt7a* and *Wnt4a* regulation allows the synchronization of the uterus and embryo development during the pre-implantation period of the mare as proposed in sheep and mice [28], [33], [34].

The AbdB-like Hoxa family includes a second class of genes differentially expressed during early pregnancy and primarily confined to the mesenchymal cell compartment during in utero development [35], [36]. Among these genes, AMCs and EDCs share the expression of *Hoxa-9*, which is specifically involved in the Müllerian duct cell patterning into oviducts, uterus, and vagina [35], as well as uterine function during early pregnancy [36], [37]. In particular, this gene is considered as one of the primary steroid hormone-responsive genes in the uterine stroma of humans and mice [35]. Its expression is regulated by factors such as ovarian steroids, interferons, and embryonic oestrogen in human [38], mouse [31], sheep [39], pig [40], and recently in equine endometrium [28]. As in humans, equine proliferation and secretion of endometrial cells, implantation of embryo, renewal of endometrial layers, and configuration of endometrial gland morphogenesis [29], [33], [41] are essential processes for the establishment of pregnancy [42] controlled by ovarian steroid hormones and local growth factors [43]. Among these, progesterone and estrogens are at the forefront of the cascade that could play a critical role in endometrial maturation and receptivity [44] and are thought to influence pre-implantation conceptus development [45], [46]. These hormones are thought to act indirectly via the endometrium. As mentioned above, progesterone mediates endometrial maturity and receptivity through specific nuclear receptors [47]. Data obtained herein demonstrate that the levels of expression of *mPR* and *PGRMC1* detected in AM and AMCs respectively, are even higher than those found in ED and EDCs. These findings may indicate the differential biological effect exerted by progesterone on the amniotic membrane and on the cells derived from it. While in ED it acts through a relatively slow ‘genomic pathway’ [45], [46], in the amniotic tissues progesterone seems to work via ligand-specific steroid receptors, including the non-classical membrane-associated receptors we analysed (*PGRMC1* and *mPR*) [48], [49].

The co-expression of *Wnt* and *Hoxa9* as well as the progesterone receptors in AMCs, suggests that these cells share similar cellular and molecular characteristics with uterine stromal cells. For some of the genes studied, our results confirm those obtained in humans, showing the same pattern of expression [50]. The loss of expression of some markers observed soon after AMCs isolation, as well as in EDCs, could depend on the culture conditions employed in this study with the consequent changes induced during the expansion.

When the expression of *FOXO1*, *SGK1*, and *TP53* was investigated the levels found in AM and AMCs were significantly lower than those observed in ED and EDCs. So far, no data has been published regarding these markers as crucial players in the process of conceptus-endometrium interaction in the equine species, and only one paper reports the link between TP53 transcriptional activity and cattle reproduction (10). Further studies are required to elucidate the role of *FOXO1*, *SGK1*, and *TP53* regulation in horses. At a molecular level, this study reinforces our previous findings regarding the immunomodulatory potential of the amniotic membrane and the cells derived from them (7) and further highlights their potential use in cell-based therapies. This observation comes from the higher levels of *PTGS2* expression found in AM and AMCs compared to their endometrial counterparts. It is important to point out that prostaglandin E_2_ not only exerts a crucial role as immunosuppressive molecules during pro-inflammatory events, but are also known to be actively involved at the onset of pregnancy as temporary luteo-protective factors [51].

To recreate the biochemical cues found during gestation, characterized by high progesterone levels, we optimized the AMCs culture system supplementing the media with progesterone. The presence of progesterone in the culture media was sufficient to preserve the expression of membrane-bound intracellular progesterone receptors and *Hoxa-9* along the 5 passages *in vitro*. Since endometrial receptivity depends on the number of progesterone receptors in epithelial and stromal cells [52], our data provide an intriguing indication of the possible application of AMCs in endometrial regeneration.

Whether the major contribution of AMCs to the healing process depends on their ability to differentiate, the immunomodulatory and trophic factors they release, or is a combination of the two mechanisms is still under debate [53], [54]. The present study also provides evidence of the role that soluble signals produced by AMCs may have in promoting EDC proliferation. Indeed, the presence of AMCs by transwell system or the effect of AMC-CM increased the EDCs proliferation.

In conclusions, the present work lays the foundation for *in vivo* studies on the potential application of AMCs in the treatment of horse uterine diseases. A substantial overlap was found between equine amnion and endometrium based on gene expression profiling, indicating that AMCs are ideal therapeutic candidates for uterine cells replenishment when scarcity or low proliferation of uterine endometrial cells is associated with pregnancy failure. Besides this, our data reinforce the idea that MSCs obtained from gestational tissues exert their beneficial effects in tissue regeneration through the release of soluble factors and indicates the conditioned media as novel therapeutic cell-free products for regenerative medicine applications.

## Supporting Information

Figure S1 Characterization of AMCs. (A) Flow cytometric analysis for the evaluation of pluripotency-associated markers (Oct-4, c-Myc and SSEA-4). Histograms represent relative number of cells vs. fluorescence intensity. Gray histograms indicate background fluorescence intensity of cells labelled with isotype control antibodies only; green histograms show positivity for the marker of interest. (B) RT–PCR analysis of mesenchymal (*CD29, CD44, CD166* and *CD105*), haematopoietic (*CD34*) specific gene expression at P1. Major histocompatibility complex (*MHC*) I and II gene expression is also reported.
(TIF)

Materials S1 (DOC)

References S1 (DOC)

Results S1 (DOC)
