# Supplementary figures and images for: Amniotic Membrane-Derived Mesenchymal Cells and Their Conditioned Media: Potential Candidates for Uterine Regenerative Therapy in the Horse
Source: PLoS One. 2014 Oct 31;9(10):e111324. doi: 10.1371/journal.pone.0111324 (PMC4216086; doi:10.1371/journal.pone.0111324)

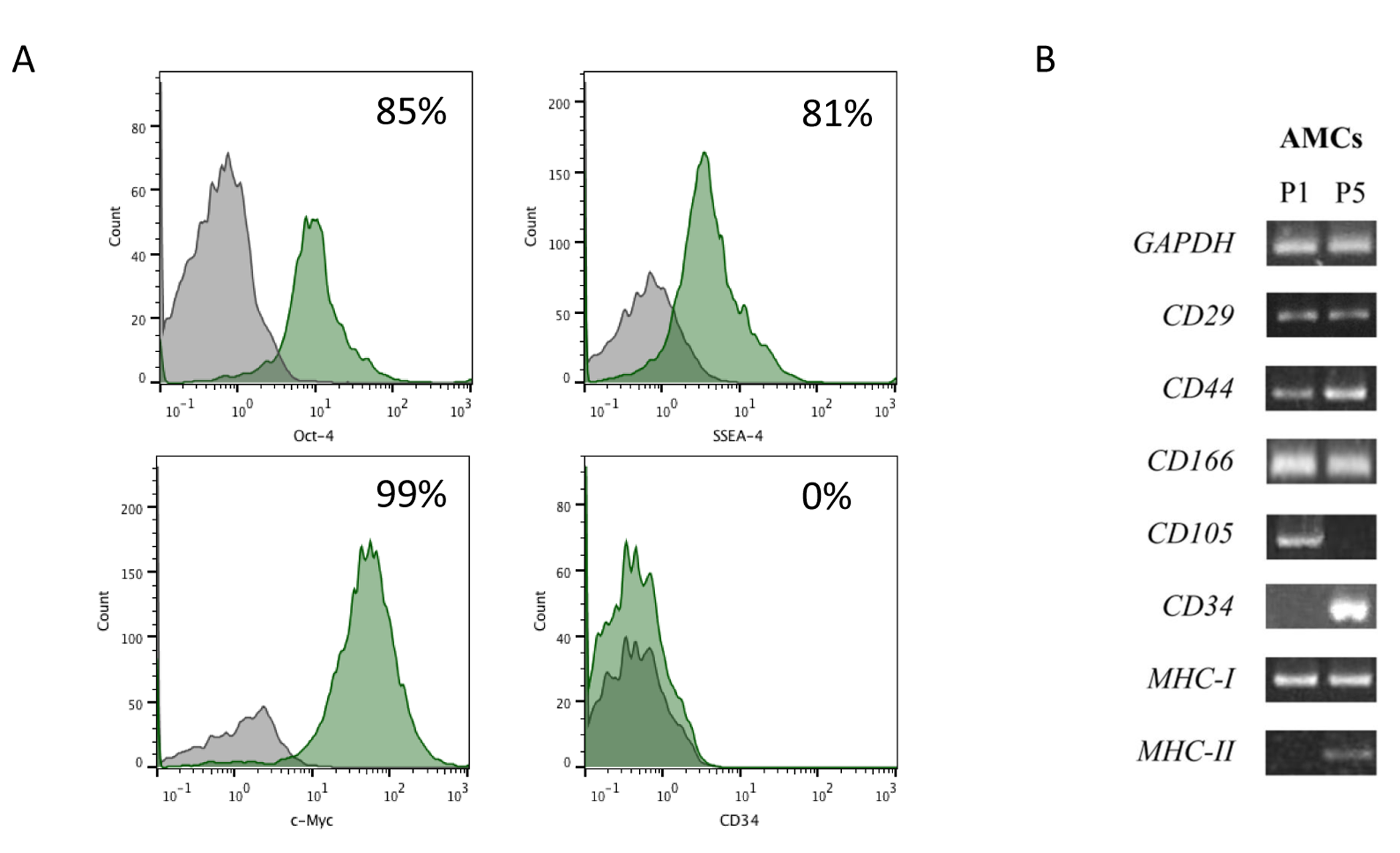

Supplement: Figure S1 — Characterization of AMCs. (A) Flow cytometric analysis for the evaluation of pluripotency-associated markers (Oct-4, c-Myc and SSEA-4). Histograms represent relative number of cells vs. fluorescence intensity. Gray histograms indicate background fluorescence intensity of cells labelled with isotype control antibodies only; green histograms show positivity for the marker of interest. (B) RT–PCR analysis of mesenchymal (CD29, CD44, CD166 and CD105), haematopoietic (CD34) specific gene expression at P1. Major histocompatibility complex (MHC) I and II gene expression is also reported. (TIF) [file pone.0111324.s001.tif]
