# Supplementary material for: Amniotic Membrane-Derived Mesenchymal Cells and Their Conditioned Media: Potential Candidates for Uterine Regenerative Therapy in the Horse
Source: PLoS One. 2014 Oct 31;9(10):e111324. doi: 10.1371/journal.pone.0111324 (PMC4216086; doi:10.1371/journal.pone.0111324)
Supplement: Materials S1 — (DOC) [file pone.0111324.s002.doc]

**MATERIALS AND METHODS**

**AMCs characterization**

**Flow cytometric analysis**

The undifferentiated nature of AMCs was previously defined by flow cytometry and molecular analysis [1-4]. Antibodies were chosen according to results obtained by Corradetti et al 2011 [5] and purchased from Abcam (Cambridge, MA, USA). Isotype-specific IgM and IgG were purchased from Abcam and Immunotools GmbH respectively. AlexaFluor-488 conjugated secondary antibodies from Invitrogen (Carlsbad, CA, USA). Tested markers included haematopoietic lineage marker (CD34), pluripotentcy markers (OCT-4, SSEA-4, and c-Myc). Briefly, AMCs (2 · 10^6^ cells ⁄ml) were labeled at passages 3 (P3) with primary antibodies in PBS 3% BSA (BDH; VWR International Ltd, Poole, UK) for 45 min at room temperature in the dark, washed in cold PBS and then incubated with secondary the secondary antibody (1:250) for 30 min at room temperature in the dark. For evaluation of nuclear markers including OCT-4 and c-Myc, cells were fixed in 0.01% paraformaldehyde (in PBS) at 4 °C for 15 min, washed in 3% BSA in PBS and treated to promote permeability for 10 min at room temperature in 1% Triton-X 100 in PBS. Cells were washed twice in ice cold PBS and analyzed using an Epics Coulter flow cytometer (Beckman Coulter-IL, Fullerton, CA, USA). The percentage of positive cells was established using FlowJo software by comparing experimental cells to control groups (isotype-specific igGs or IgM).

**Gene expression**

Expression of specific MSC (CD44, CD29, CD105 and CD166) and haematopoietic (CD34) markers was investigated by RT–PCR analysis. Total RNA was extracted at P1 and P5 from equine AMCs, using TrizolW reagent (Invitrogen), followed by DNAse treatment according to the manufacturer’s specifications. RNA concentration and purity were measured using a NanoDrop spectrophotometer (NanoDropW ND1000). cDNA was synthesized from 200 ng total RNA, using the iScript retrotranscription kit (Bio-Rad Laboratories, Hercules, CA, USA). Conventional PCR was performed in a 25ml final volume with DreamTaq DNA Polymerase (Fermentas, St. Leon Rot, Ger- many). Equine-specific oligonucleotide primers were designed and used as previously reported [1].
