# Supplementary material for: Amniotic Membrane-Derived Mesenchymal Cells and Their Conditioned Media: Potential Candidates for Uterine Regenerative Therapy in the Horse
Source: PLoS One. 2014 Oct 31;9(10):e111324. doi: 10.1371/journal.pone.0111324 (PMC4216086; doi:10.1371/journal.pone.0111324)
Supplement: References S1 — (DOC) [file pone.0111324.s003.doc]

**REFERENCES**

1. Lange-Consiglio A, Corradetti B, Bizzaro D, Magatti M, Ressel L, et al. (2012). Characterization and potential applications of progenitor-like cells isolated from horse amniotic membrane. J Tissue Eng Regen Med 6: 622-635.

2. Lange-Consiglio A, Corradetti B, Meucci A, Bizzaro D, F Cremonesi (2013a) Characteristics of equine mesenchymal stem cells derived from amnion and bone marrow: *in vitro* proliferative and multilineage potential assessment Equine Vet J 45(6): 737-744.

3. Lange-Consiglio A, Tassan S, Corradetti B, Meucci A, Bizzaro D, et al. (2013b) **Investigating the potential of equine mesenchymal stem cells derived from amnion and bone marrow in equine tendon diseases treatment in vivo.** Cytotherapy 15: 1011-1020.

4. Lange-Consiglio A, Rossi D, Tassan S, Perego R, Cremonesi F, et al. (2013c) Conditioned medium from horse amniotic membrane-derived multipotent progenitor cells: immunomodulatory activity in vitro and first clinical application in tendon and ligament injuries in vivo. Stem Cells Dev 22(22): 3015-3024.

5. Corradetti B, Lange-Consiglio A, Barucca M, Cremonesi F, Bizzaro D (2011) Size-sieved subpopulations of mesenchymal stem cells from intervascular and perivascular equine umbilical cord matrix. Cell Proliferation 44: 330-342.
