# Supplementary material for: Amniotic Membrane-Derived Mesenchymal Cells and Their Conditioned Media: Potential Candidates for Uterine Regenerative Therapy in the Horse
Source: PLoS One. 2014 Oct 31;9(10):e111324. doi: 10.1371/journal.pone.0111324 (PMC4216086; doi:10.1371/journal.pone.0111324)
Supplement: Results S1 — (DOC) [file pone.0111324.s004.doc]

**RESULTS**

**Figure S1a** shows the immunophenotyping profile of AMCs at passage 3, with the percentage of cells found positive for each marker. AMCs were negative for the hematopoietic marker CD34 but the number of cells positive for the pluripotency-associated markers (Oct-4, SSEA-4, and c-myc) was consistently high (close to 90% for each marker). At the transcriptional level, AMCs expressed MSC-associated markers (*CD29*, *CD44*, *CD166* and *CD105*) at P1 and P5. The expression of the *CD34* marker was not registered at P1 but was present at P5. AMCs expressed *MHC-I* at P1 and P5 and lacked *MHC-II* expression at P1, but began to express this marker at P5 (**Figure S1b**).
